# New insights into inflammatory osteoclast precursors as therapeutic targets for rheumatoid arthritis and periodontitis

**DOI:** 10.1038/s41413-023-00257-w

**Published:** 2023-05-22

**Authors:** Emilie Hascoët, Frédéric Blanchard, Claudine Blin-Wakkach, Jérôme Guicheux, Philippe Lesclous, Alexandra Cloitre

**Affiliations:** 1 Nantes Université, Oniris, Univ Angers, CHU Nantes, INSERM, Regenerative Medicine and Skeleton, RMeS, UMR 1229, F-44000 Nantes, France; 2 Université Côte d’Azur, CNRS, LP2M Nice, France

**Keywords:** Bone, Pathogenesis, Dental diseases

## Abstract

Rheumatoid arthritis (RA) and periodontitis are chronic inflammatory diseases leading to increased bone resorption. Preventing this inflammatory bone resorption is a major health challenge. Both diseases share immunopathogenic similarities and a common inflammatory environment. The autoimmune response or periodontal infection stimulates certain immune actors, leading in both cases to chronic inflammation that perpetuates bone resorption. Moreover, RA and periodontitis have a strong epidemiological association that could be explained by periodontal microbial dysbiosis. This dysbiosis is believed to be involved in the initiation of RA via three mechanisms. (i) The dissemination of periodontal pathogens triggers systemic inflammation. (ii) Periodontal pathogens can induce the generation of citrullinated neoepitopes, leading to the generation of anti-citrullinated peptide autoantibodies. (iii) Intracellular danger-associated molecular patterns accelerate local and systemic inflammation. Therefore, periodontal dysbiosis could promote or sustain bone resorption in distant inflamed joints. Interestingly, in inflammatory conditions, the existence of osteoclasts distinct from “classical osteoclasts” has recently been reported. They have proinflammatory origins and functions. Several populations of osteoclast precursors have been described in RA, such as classical monocytes, a dendritic cell subtype, and arthritis-associated osteoclastogenic macrophages. The aim of this review is to synthesize knowledge on osteoclasts and their precursors in inflammatory conditions, especially in RA and periodontitis. Special attention will be given to recent data related to RA that could be of potential value in periodontitis due to the immunopathogenic similarities between the two diseases. Improving our understanding of these pathogenic mechanisms should lead to the identification of new therapeutic targets involved in the pathological inflammatory bone resorption associated with these diseases.

## Introduction

Rheumatoid arthritis (RA) is a systemic autoimmune disease characterized by chronic inflammation of the joints leading to increased bone resorption and multiple articular disabilities. It affects 0.5%–1.0% of the world’s population.^1^ A combination of genetic and environmental factors leads to the breakdown of immune tolerance in mucosal surfaces, including the periodontium.^2^ Posttranslational modification of proteins results in the generation of neoepitopes, which can lead to the formation of anti-modified protein antibodies such as anti-citrullinated peptide antibodies (ACPAs) and rheumatoid factor (RF).^3^ In the joints, the binding of ACPAs to neoepitopes and the formation of immune complexes containing RF lead to a vicious cycle of tissue damage.

Periodontitis is a multifactorial chronic inflammatory disease resulting from a dysbiotic biofilm leading to the destruction of the tooth-supporting bone, i.e., the alveolar bone.^4^ It is a highly prevalent condition, since 42% of the US population aged 30 or older is affected by periodontitis and 59.8% of those over 65.^5^ Moreover, its severe form, characterized by major alveolar bone loss, is the sixth most common disease worldwide.^6^ Despite well-managed mechanical treatment and rigorous oral hygiene, a recurrence rate of up to 26% is reported.^7^ A strong association between gram-negative bacteria and periodontitis has been reported, particularly with the red complex triad: *Porphyromonas gingivalis (P. gingivalis)*, *Tannerella forsythia*, and *Treponema denticola*.^8^ Mostly, periodontal destruction is the consequence of the host’s exacerbated response to bacterial stimuli through an inflammatory cascade.^9^ Therefore, as in RA, the immune response in periodontitis is the major determinant of susceptibility to disease.

The resorption of bone tissue is a unique characteristic of osteoclasts (OCs), which are multinuclear cells formed by the differentiation and fusion of osteoclast precursors (OCPs) from the monocyte (MN)/macrophage (MP) hematopoietic lineage.^10^ Recently, mouse inflammatory osteoclasts (iOCs) capable of inducing inflammatory responses have been described.^11–13^ iOCs participate in inflammation by producing proinflammatory cytokines, presenting antigens, and inducing the activation of T cells in the bone marrow. Such OCs have not yet been described in humans. In RA in mouse and human studies, different OCPs have been described, such as classical^14^ MNs, MN-derived immature dendritic cells,^15^ and arthritis-associated osteoclastogenic macrophages (AtoMs).^16^ However, their contribution as progenitors of OCs with inflammatory properties remains to be demonstrated, at least for some of them.

The aim of this comprehensive review is to synthesize knowledge on OCs and their precursors in inflammatory conditions with special attention to the recent new data related to RA and potential involvement in periodontitis pathogenesis.

## Part I. Similarities between rheumatoid arthritis and periodontitis

### Common genetic and environmental risk factors

RA and periodontitis share genetic and environmental risk factors, including the presence of the HLA-DRB1 shared epitope,^17,18^ smoking, poor nutrition, socioeconomic status, and psychological factors.^3^ A strong epidemiological association is also found between RA and periodontitis. A recent meta-analysis showed that patients with periodontitis are 69% more likely to develop RA than healthy patients.^19^

In murine models, the induction of experimental periodontitis exacerbated clinical signs of arthritis with increased bone resorption.^20^ It also increased serum RF and gingival ACPA levels.^21^ Antibiotic treatment of mice with experimental periodontitis rendered these animals refractory to the induction of collagen-induced arthritis.^22^ Finally, in a model of periodontal disease-induced arthritis, chronic oral exposure to *P. gingivalis* induced severe periodontitis, leading to elevated levels of circulating anti-CCP2, IL-17, and CXCL1 as well as subsequent synovial inflammation and bone destruction.^23^

Patients exposed to *P. gingivalis* had a greater risk of developing RA,^24^ and RA patients had a dysbiotic subgingival microbiome with an increase in *P. gingivalis* compared to healthy controls.^25^ RA patients with periodontitis had a significantly higher disease activity score (DAS)^26^ and a significant increase in blood neutrophil extracellular trap (NET)^27^ concentration than RA patients without periodontitis. In addition, a high blood level of anti-*P. gingivalis* antibodies were associated with an increase in blood ACPAs and erythrocyte sedimentation rate and a high prevalence of erosive lesions in RA.^28,29^ Moreover, patients with severe RA had more severe periodontitis^30^; increased periodontal attachment loss correlated with increased DAS28-CRP score,^31^ and elevated blood MMP-3 levels.^30^ Of interest, local periodontal treatment reduced the severity of RA in patients and was associated with a decrease in blood levels of NETs,^27^ IL-1β, IL-8,^27^ TNFα,^28^ RANKL,^26^ ACPAs and, in particular, anti-cyclic citrullinated peptide (anti-CPP)^28^ and a decrease in anti-*P gingivalis* antibody levels.^28^ Thus, it induced a decrease in the DAS score^26–28^ CRP^26,28^ and erythrocyte sedimentation rate.^28^

### Immunopathogenic similarities

**Fig. 1:**
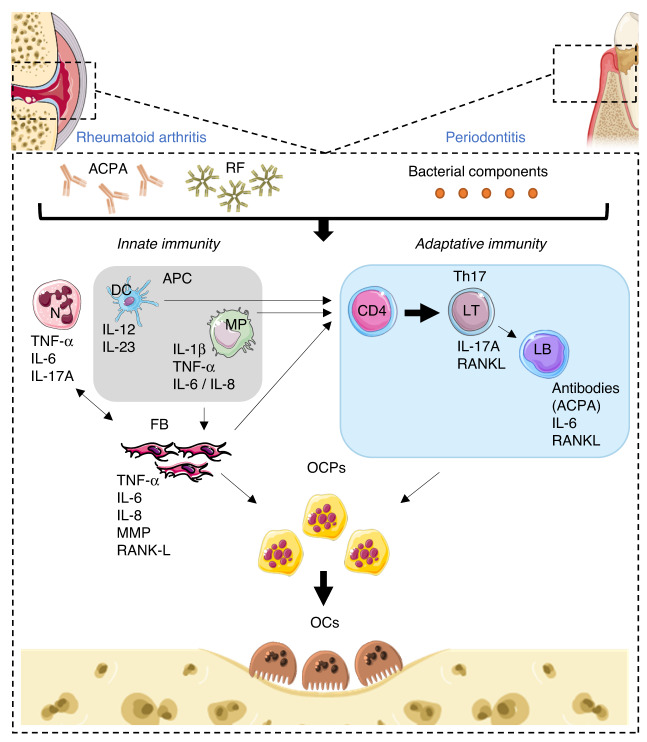
Immunopathogenic similarities between rheumatoid arthritis and periodontitis. In response to anti-citrullinated peptide antibodies (ACPA) or rheumatoid factor (RF) in rheumatoid arthritis or to bacterial components in periodontitis, innate immune cells (macrophages (MP), dendritic cells (DC), neutrophils (N)) infiltrate the synovium or the inflammatory periodontium. This induces the synthesis of pro-inflammatory cytokines and the stimulation of fibroblasts (FBs). Activated fibroblasts, in turn, produce pro-inflammatory cytokines, matrix metalloproteinase (MMP), and RANKL. Naive CD4^+^ T cells are then activated by antigen-presenting cells (MPs and DCs) and proliferate and differentiate. Depending on the cytokine environment, the T helper cell response can involve Th1 proinflammatory cells, Th2 anti-inflammatory cells, or Th17 effector cells. Th1 and Th17 cells activate B cells, which then also produce RANKL. These sources of RANKL promote the differentiation of osteoclast precursors (OCPs) and lead to bone resorption by osteoclasts (OCs). In addition, there is an autoamplification loop by regulations between different actors. MPs and FBs recruit neutrophils via an IL-8 gradient. Th1 and Th17 cells as well as B cells participate in the regulation of innate cells (N recruitment, MP activation), and FBs stimulated by Th17 cells respond by secreting IL-6, which promotes the differentiation of naive CD4^+^ T cells and B cells

RA and periodontitis share immunopathogenic similarities (Fig. 1). In RA, the generation of neoepitopes in the periodontium or lymph nodes leads to the formation of ACPAs. Locally in the joint, the binding of ACPAs to citrullinated epitopes and the formation of immune complexes containing RF induce local inflammation. In periodontitis, due to bacterial dysbiosis, pathogen-associated patterns (PAMPs) such as lipopolysaccharide (LPS) from gram-negative bacteria are recognized by host innate immune cells.^3^ These cells infiltrate the synovium or the periodontium and induce tissue damage through the release of proinflammatory cytokines and tissue-degrading enzymes. In RA and periodontitis, inflammation is initially mediated by the activation of resident cells (epithelial cells and fibroblasts), MPs, and dendritic cells (DCs).^9,32^ Subsequently, activated antigen-presenting cells initiate self-antigen-specific T and B-cell responses in the lymph nodes and local tissues. Finally, in both diseases, increased bone resorption is observed, i.e., articular and alveolar resorption.

#### Innate immunity

Resident cells (epithelial cells, fibroblasts, MPs) act as an immune barrier. Oral epithelial cells in periodontitis are the first to initiate the innate immune response by producing proinflammatory cytokines such as IL-1β, IL-6, TNF-α, and IL-8 (or CXCL8), a chemokine attractant for neutrophils.^33^ In vitro, they have been shown to produce receptor activator of nuclear factor kappa B ligand (RANKL) in the basal state at a level sufficient for osteoclastic differentiation and activation in a coculture system with murine bone marrow (BM) MP.^34^

In RA and periodontitis, fibroblasts stimulated by activated immune cells^32^ produce various mediators of inflammation, cytokines (IL-1β, IL-6, TNF-*α*), chemokines (IL-8, CXCL10, CXCL11, CCL20), prostaglandin E2,^35^ matrix metalloproteinases (MMPs),^32^ and RANKL.^32,36^ Fibroblasts contribute directly to local joint damage, but they can also migrate between joints in RA, promoting inflammation in other joints and demonstrating the symmetric nature of the disease.^37^

Activated MPs in RA produce proinflammatory cytokines (IL-1β, IL-6, TNF-α) that contribute to increased inflammation by recruiting and activating other innate immune cells, such as neutrophils, to the site of synovitis.^32^ Their infiltration appeared to be a prerequisite for B-cell activation and plasma cell development.^38^ Langerhans cells (CD207^+^) of the epithelium in periodontitis were significantly decreased compared to healthy patients, which might be due to a massive migration of these cells to the lymph nodes to present antigen, ensuring the transition from innate to acquired immunity.^39^ In RA and periodontitis, inflammatory MPs are essentially phagocytic cells that eliminate pathogens and cellular debris and may also present antigens to enable the activation of acquired immunity.^40^

Neutrophils are recruited in large numbers to the inflammatory synovium or periodontium by a chemotactic gradient (linked mainly to IL-8) due to the host inflammatory response.^40^ Activated neutrophils have phagocytic ability to eliminate pathogens, and they also produce an arsenal of proteases such as MMPs, pro-inflammatory cytokines such as IL-1, IL6, TNF-α, and IL-17A, and chemokines that contribute to joint destruction.^32,41^ Individuals with congenital deficiencies in neutrophil number or recruitment develop severe periodontitis, suggesting that neutrophils are mandatory for periodontal tissue homeostasis.^42^ In addition, neutrophil hyperactivity persists after successful periodontal treatment.^43,44^

DCs migrate to the inflammatory synovium or periodontium.^45^ DCs enhance the inflammatory process, leading to the activation of various immune cells and increased tissue destruction.^32,39^ In addition, ACPA-positive RA patients had a higher number of plasmacytoid DCs (pDCs) that could promote the production of self-antibodies via the expression of anti-apoptotic B-cell-activating factor (BAFF).^46^ Finally, DCs have a critical function in the regulation of immune responses by taking up, processing, and presenting antigens to naive T cells.^32^ In RA patients, recruitment of DCs in the synovium reduces the frequency of conventional DCs (cDCs) and plasmacytoid DCs (pDCs) in the blood.^47^ In contrast to steady-state synovium and lymphoid organs, the synovium of RA patients also contains inflammatory DCs (CD14^+^ CD1a^+^ CD1c^+^), which are derived from the differentiation of circulating monocytes during inflammation.^48^ They produce IL-12 and IL-23, which promote antigen-specific Th17 responses, resulting in imbalances between Th1, Th2, and Th17 responses.^46,48^ Furthermore, synovial DCs contribute to the maintenance of inflammation by expressing lower levels of CCR7, resulting in reduced rates of emigration of mature DCs from inflamed tissues and maintenance of local inflammation.^49^ In patients with periodontitis, the level of pDCs (CD123^+^) is increased, while that of cDC2 (CD1c^+^, DCs predominant in the gingival tissue) and cDC1 (CD141^+^, present in the lamina propria (connective tissue under the epithelium)) remains unchanged compared to healthy patients.^39^ The increase in pDCs (CD123^+^) in gingival samples is accompanied by increased expression of the proinflammatory cytokines IL-1β, IFN-α, and IFN-γ, while the anti-inflammatory cytokine IL-10 is suppressed.^39^

#### Adaptive immunity

In early RA, half of the patients showed a lympho-myeloid pathotype characterized by infiltration of B and T lymphocytes and myeloid cells in the synovium.^38^ In periodontitis, the lymphocyte population changes during the course of disease. In the early stage of gingivitis that precedes the periodontitis stage, T cells predominate, but once periodontitis is established, B cells and plasma cells constitute the majority.^50^ Activated T cells that migrate to the synovium and periodontium interact locally with MPs, DCs, fibroblasts, and resident OCs.^32^ CD8^+^ T cells recognize and destroy infected cells. Naive CD4^+^ T cells are activated by antigen-presenting cells and proliferate and differentiate into T helper cells and memory CD4^+^ T cells. Depending on the cytokine environment, the T helper cell response can involve Th1 pro-inflammatory cells (IFN-γ), Th2 anti-inflammatory cells (IL-13, IL-4, IL-6, IL-5), Th17 effector cells (IL-17, IL-22), or T regulatory (Treg) suppressive cells (TGF-β, IL-10, IL-35).^51^ In RA, it has been suggested that Th17 cells may play an important role in the early stages of the disease, while in the later stages, Th1 cell differentiation into cytotoxic CD4^+^ T cells may lead to both direct tissue damage and the production of proinflammatory cytokines.^52^ In periodontitis, Th1 cells are more important in the early stage, while Th2 cells are more numerous in the late stage,^53^ in which Th17 cells are also identified.^54^

In both diseases, Th1 cells release IL-2, IFN-γ, and TNF-β, leading to the activation of MPs and B cells, which trigger and perpetuate inflammatory responses both in the synovium and in the periodontium.^52,53^ Th17 cells, induced by the cytokines IL-6, IL-1β, IL-21, TGF-β, and IL-23, recruit neutrophils, activate B cells, and promote osteoclastogenesis via the production of RANKL and IL-17A.^55,56^ In addition, there is an autoamplification loop, since the fibroblasts stimulated by Th17 cells respond by releasing IL-6, which promotes the differentiation of CD4^+^ T cells.^55^ In RA, it has been shown that the inflammatory environment can contribute to the dysfunction of Treg cells and their differentiation into pathological T cells. Indeed, CD4^+^CD25^+^Foxp3^+^ Treg cells with the potential to convert into pathogenic Th17 cells accumulate in the inflamed synovium or periodontium.^57,58^ Thus, the Th17/Treg balance is altered in both diseases.

B cells are responsible for the humoral-mediated adaptive response directed against extracellular antigens. In RA, RF and ACPAs are the two main types of self-antibodies defining a patient as “seropositive”.^59^ The presence of RF and ACPAs has been correlated with a 40% risk of disease onset.^60^ ACPAs and RF are found in 60%–80% and 69% of RA patients, respectively, and their specificity for disease is 85%–99% and 60%–85%, respectively.^32^ In periodontitis, plasma cells secrete antibodies that are specifically directed against incriminating bacterial antigens.^40^ In both diseases, B cells have other functions. They participate in the regulation of innate and adaptative immunity through the release of cytokines (including elevated levels of IL-1β and IL-6)^61,62^ and RANKL.^62,63^ Therefore, B-cell deletion therapy in RA (rituximab) induced a reduction in IL-1β levels in gingival crevicular fluid in patients with periodontitis.^64^ In contrast to proinflammatory B-cell responses, regulatory B cells (Breg) exert immunosuppressive functions by producing anti-inflammatory cytokines such as IL-10.^62^ In RA patients, Bregs can inhibit disease progression by inducing the production of IFN-γ and IL-21 by T cells while reducing the production of ACPAs.^62^ The decrease in the number of Bregs is correlated with the increase in disease activity. In *P. gingivalis*-associated ligature-induced experimental periodontitis, the adoptive transfer of Breg cells (CD1d^hi^CD5^+^) induced a gingival decrease in the production of RANKL, TNF-α, and IL-1β and an increase in the production of IL-10, which inhibited periodontal bone loss.^65^

#### Major cytokines involved in the inflammatory cascade and bone resorption

In RA and periodontitis, adaptive and innate immune cells promote the release of proinflammatory cytokines (TNF-α, IL-6, IL-17A, IL-1β, RANKL), which have an important role in establishing and maintaining inflammation.^66^

TNF-α is one of the most important mediators of joint and periodontal inflammation.^32,66^ It induces the differentiation of MN/MP lineage cells into OCs,^67^ increases RANKL expression by osteoblasts, and causes bone resorption.^68^ Another important role of TNF-α is to induce the production of other inflammatory cytokines, such as IL-1β and IL-6, which attract leukocytes and promote an inflammatory environment in the synovium.^69^ In RA patients with periodontitis, the level of TNF-α is correlated with the severity of periodontal disease,^3^ and anti-TNF-α treatment improved both diseases.^70^

IL-6 is an important cytokine for both innate and adaptive immunity and has a role in the differentiation of T-helper cells, Th17 cells, and B cells.^71^ It is also a potential inducer of OC differentiation independently of RANKL,^72^ and blocking IL-6R inhibits OC formation. IL-6 is produced by fibroblasts, MNs, and T and B cells.^62,71^ In RA patients, the concentration of IL-6 in the blood and synovium is increased compared to that in healthy donors and is associated with joint damage.^71^ In RA treatment, the IL-6 receptor inhibitors tocilizumab and sarilumab are used as monotherapy or in combination with methotrexate.^71^

IL-17A promotes the production of the proinflammatory cytokines IL-6, IL-8, GM-CSF,^73^ MMP-1,^74^ and RANKL,^75^ leading to OC differentiation and bone resorption. IL-17A is mostly produced by Th17 cells but also by neutrophils, γδ T cells, and natural killer T cells.^76^ In murine models, oral infection with *P. gingivalis* prior to arthritis induction caused aggravation of arthritis associated with an increase in Th17 cells in the blood and in the synovium and neutrophil infiltration.^77,78^ Such increases were not reported in IL-17-deficient mice, highlighting the importance of Th17 cells in the pathogenesis of periodontitis and RA.^78^ However, the role of IL-17A in RA remains debatable, as therapeutic targeting of IL-17A or IL-17R has been shown to be less effective than placebo in TNF-α-resistant or methotrexate-resistant RA despite a significant benefit in psoriatic arthritis.^79^

IL-1β is a proinflammatory cytokine secreted by innate immune cells (MNs, MPs, DCs, and neutrophils).^80^ IL-1β is crucial for T-cell differentiation,^81^ and it also activates the RANK-RANKL pathway, resulting in increased osteoclastogenesis.^80^ In addition, IL-1β (like TNF-α) amplifies the effects of IL-17A.^3,82^ However, in RA, anakinra (an IL-1 inhibitor) is less effective than other disease-modifying anti-rheumatic drugs (DMARDs) and is rarely used in clinical practice.^83^ In a ligature-induced periodontitis rat model, intrapapillary injections of IL-1Ra significantly inhibited gingivitis and the loss of alveolar bone compared to controls.^84^ However, these results have not been confirmed by human clinical data.

RANKL activates the differentiation and maturation of OCs and leads to bone resorption. The sources of RANKL are numerous and include immune cells (Th17 cells, MPs, activated DCs and B cells) and activated resident cells (osteoblasts, osteocytes, fibroblasts, and epithelial cells).^32^ RANKL knockout mice are protected against serum transfer-induced arthritis.^85^ In periodontitis, *P. gingivalis* LPS increased RANKL expression by oral epithelial cells^86^ and osteoblasts via TLR2, which led to the differentiation and activation of OCs.^87^ Furthermore, stimulation of bone marrow-derived MPs by *P. gingivalis* induced bone resorption, but the mechanism by which MP differentiation in OCs is increased or RANKL production is enhanced has not been fully deciphered.^87^ Interestingly, some therapies targeting inflammatory cytokines and B cells have shown clinical efficacy in both RA and periodontitis patients, thus strengthening the immunopathogenic link between these diseases (Table 1).^32,71,79,88–95^

**Table 1:** Targeted therapy that has shown clinical efficacy in rheumatoid arthritis or periodontitis patients

Disease	Anti-TNF	Anti-IL6R	Anti-IL17A	T-cell costimulation inhibitor	Anti-B-cell
Rheumatoid arthritis	Infliximab^32^ Adalimumab^32^ Etanercept^32^ Certolizumab^32^	Tocilizumab^71^ Sarilumab^71^	Secukinumab^,^^79,88^ Ixekizumab^79,88^	Abatacept^89^	Rituximab^32^
Periodontitis	Infliximab^90,91^ Adalimumab^92^ Etanercept^92^ Golimumab^92^	Tocilizumab^93^	Secukinumab^94^	NR	Rituximab^95^

*NR* not reported

### A common etiology?

**Fig. 2:**
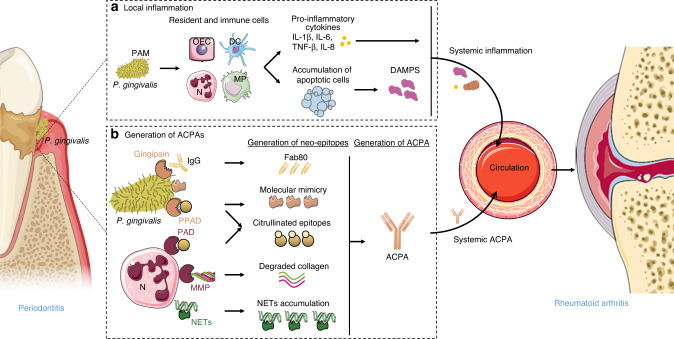
Common etiology between rheumatoid arthritis and periodontitis. During periodontitis, different mechanisms can promote the development of rheumatoid arthritis**. a** In inflamed periodontium, pathogen-associated patterns (PAMPs) from periodontal pathogens such as *P. gingivalis* are recognized by resident and immune cells (oral epithelial cells (OEC), dendritic cells (DC), neutrophils (N), and macrophages (MP)). These cells then secrete proinflammatory cytokines such as IL-1β, TNF-α, IL-6, and IL-8. In parallel, the inhibition of bacterial phagocytosis and complement factors leads to an accumulation of apoptotic cells and secondary necrosis, which induce the release of damage-associated molecular patterns (DAMPs). This leads to local and then systemic inflammation via the hematogenous dissemination of periodontal bacteria and inflammatory mediators. **b** Periodontal bacterial dysbiosis generates neo-epitopes that promote the appearance of anti-citrullinated protein antibodies (ACPAs) in the gingival tissue. *P. gingivalis* can generate neoepitopes by its gingipain, which cleaves human IgG to release Fab80 fragments, via molecular mimicry with human self-antigens, and by its peptidylarginine deiminase (PPAD), which citrullinates proteins. PNNs generate neoepitopes via their peptidylarginine deiminases (PAD), which also citrullinate proteins, through the matrix metalloproteinase (MMP) that induces collagen degradation and by generation of neutrophil extracellular traps (NETs). The presence of neoepitopes leads to ACPA generation in the periodontal tissue and then in the systemic circulation, which can induce an autoimmune response in the synovial tissue and rheumatoid arthritis

The relationship between RA and periodontitis may be explained by a common etiology (Fig. 2). It has been argued that RA is triggered by interactions between the periodontal microbiome and the host in two main ways.^96^ First, systematic chronic inflammation, bacteremia and repeated translocation of microorganisms from the oral or intestinal microbiome in some synovia induce the formation of ACPAs and durable epigenetic modifications in long-lived synovial immune cells.^25^ Second, the generation of neoepitopes or the release of cellular contents in the periodontal tissue could induce the production of antibodies responsible for the subsequent immune response in the synovium. The mechanisms are detailed below.

#### Bacteremia, oropharyngeal translocation and bone resorption

Periodontal infection induces local inflammation followed by systemic chronic inflammation via dissemination of bacteria and inflammatory mediators in the bloodstream but also via oropharyngeal and orodigestive translocation of periodontopathogens.

##### Bacteremia and periodontal inflammatory mediators

Aggregation of bacteria in a biofilm causes local but also systemic inflammation. Patients with severe periodontitis have higher blood levels of proinflammatory mediators (such as IL-1β, IL-6, C-reactive protein, and fibrinogen) and neutrophils than healthy donors.^43^ This systemic inflammation is attenuated by local periodontal treatment, such as scaling or root planning. Transient oral bacteremia occurs after tooth brushing, flossing, chewing, or invasive dental treatment. Multiple clinical studies have detected genomic DNA of periodontal bacteria in tissues distant from the oral cavity; however, there is little evidence of their presence in a viable state.^43^ Periodontal bacteria, such as *P. gingivalis*, have also been detected by 16 S ribosomal DNA sequencing in blood DCs^43^ and by immunohistochemistry in atherosclerotic plaques from patients with periodontitis.^43,97^ Since *P. gingivalis* can survive 24 h in DCs in vitro,^97^ it could spread to distant sites. Several studies have demonstrated increased levels of *P. gingivalis* DNA in blood and synovial fluid in RA patients compared to healthy patients.^25^ RA patients who carried the HLA DRB1*04 allele had increased levels of *P. gingivalis* DNA in synovial tissue compared to healthy patients.^98^ In particular, *P. gingivalis* DNA has been shown to stimulate MPs and fibroblasts to induce the secretion of proinflammatory cytokines.^98^ Berthelot et al. reported that in patients with RA and periodontitis, identical bacterial clones were detected in synovial fluid and dental plaque.^25^ In addition, since the synovium is not completely sterile, there may be a symbiotic state with the presence of nonreplicating bacteria that do not induce an immune response.^25^ This equilibrium could be disrupted by metabolic reprogramming of the cells and/or excessive translocation leading to the generation of NETs by neutrophils. NETs are decondensed chromatin fibers with histones, antimicrobial proteins, and cytoplasmic proteins released during NETosis.^99^ Although their antimicrobial function is beneficial, uncontrolled formation of NETs or their delayed clearance is associated with several autoimmune diseases, including RA.^99^ In RA patients, serum ACPAs react with NET histones, and neutrophils have an increased production of NETs, which is positively correlated with disease activity.^100^ Furthermore, the danger signals delivered by pathobionts to synovial tissue resident macrophages or mesenchymal stem cells could induce epigenetic changes in these cells over time and induce durable arthritis.^25^ In addition, the osteoclastic potential of BM cells from long bones and peripheral blood cells is increased following chronic subcutaneous infection of mice with *P. gingivalis*.^101^ The presence of circulating *P. gingivalis* increased systemic IL-6 levels, which promoted the differentiation of BM osteoclastic lineage cells into CD11b^+^ c-fms^+^ Ly6C^hi^ OCPs rather than inflammatory MNs or MPs.^101^ Then, these OCPs move to sites of bone resorption to participate in osteoclastogenesis in response to locally produced RANKL. Differentiation of BM cells into OCPs induced by *P. gingivalis* links periodontitis to other inflammatory diseases, such as RA.^101^

##### Oropharyngeal translocation of periodontopathogens

Oral bacteria, through oropharyngeal translocation, could lead to intestinal dysbiosis and thus induce local and remote systemic inflammation with increased intestinal permeability and translocation of various other bacteria to the synovium.^25,102^ Several studies have shown that the composition of intestinal microbiota is different in RA patients compared to healthy people,^103^ and microbiome analysis of treatment-naive RA patients revealed a higher transmissibility of bacteria from the oral cavity to the gut for all taxa.^25^ In a collagen-induced arthritis (CIA) murine model, analysis of the gut microbiome showed an increase in the proportion of *Firmicutes* and a decrease in *Bacteroidetes*.^103^ Elimination of the intestinal microbiota with broad-spectrum antibiotics led to a reduction in Th1 and Th17 cells in popliteal lymph nodes and a decrease in the arthritis severity score.^103^ In another study, oral administration of *P. gingivalis* before induction of CIA caused an aggravation of arthritis with increased IL-17 serum levels and a change in the gut microbiome with an increase in the *Firmicutes*/*Bacteroidetes* ratio.^104^ Furthermore, a meta-analysis showed that patients with inflammatory bowel disease had a higher risk of periodontitis than healthy patients.^102^ The oral and gut microbiomes may therefore play an active role in the development of RA.^96^

##### Periodontopathogenic bacteria and bone resorption

The microorganisms of bacterial dysbiosis, and more specifically *P. gingivalis*, thus seem to be strongly involved in the generation and maintenance of RA. In addition, *P. gingivalis* also plays a role in the induction of bone resorption, a hallmark of RA and periodontitis, in several ways. For example, studies have shown that it promotes the osteoclastogenic activity of BM cells and peripheral cells by inducing their differentiation into OCPs via IL-6.^101^ *P. gingivalis* also increased RANKL expression by oral epithelial cells^86^ and osteoblasts via TLR2^87^ and promoted the survival of OCs via the regulation of survivin, an anti-apoptotic factor.^26^

#### Anti-citrullinated protein antibodies in gingiva

Periodontal pathogens can induce the generation of citrullinated neoepitopes, leading, in turn, to the generation of ACPAs. These ACPAs then react with citrullinated peptides in the synovium, which may have formed after a traumatic event in the joint.^3^ ACPAs are present in approximately 70% of RA patients and are specific to the disease.^3^ ACPAs are detectable in the circulation years before the onset of any clinical symptoms of RA.^3,43^ Citrullinated neoepitopes are recorded in greater abundance in the inflamed periodontium than in healthy tissue,^105^ and the proportion of ACPAs is increased in the blood of patients with periodontitis compared to healthy controls.^28^ These results support the hypothesis that the initial loss of immune tolerance to citrullinated proteins is likely to be a consequence of an inflammatory event remote from the joint. Their four main sources in periodontitis are described below.

First, periodontal pathogens increase the activity of human peptidyl arginine deiminases (PADs), leading to the rapid and spontaneous generation of citrullinated epitopes.^3^ The presence of the human PADs PAD2 and PAD4 (and their products) in periodontitis patients supports this hypothesis.^106^ In parallel to these human PADs, *P. gingivalis* expresses its own PADs (PPADs), which can citrullinate fibrinogen, α-enolase, and vimentin in periodontal tissue and then generate citrullinated peptides and protein fragments inducing the systemic production of ACPAs that cause autoimmunity in RA.^107^ Several authors have demonstrated that PPAD from *P. gingivalis* is required to aggravate arthritis in CIA mice.^29^ PPAD activity in the periodontium is increased both in RA patients regardless of their periodontal status and in RA-free patients affected by periodontitis.^108^ Furthermore, citrullinated proteins in gingival connective tissue appeared similar to those found in synovial tissue,^28^ including citrullinated PPAD peptide, indicating that CPP3 and CPP3^+^ B cells were increased in patients with early RA and with periodontitis, both in the gingiva and in the synovial membrane.^109^

The remnant epitope generates autoimmunity (REGA) model presents another source of neoepitopes. Phagocytes secrete cytokines and proteases that cause extracellular proteolytic degradation of proteins into residual fragments containing immunodominant epitopes.^3^ In RA, neutrophils release MMP-8, which then catalyzes collagen degradation.^110^ Gingival crevicular fluid from periodontitis patients, an inflammatory exudate collected in the periodontal pocket, also exerts strong proteolytic activity that could lead to the generation of immunodominant epitopes.^111^ Finally, in vivo, lysine-specific gingipain cleaves human IgG to release Fab fragments, which are recognized as neo-epitopes by autoantibodies.^112^

Some antigens expressed by *P. gingivalis* are structurally similar to human self-antigens and can cross-react with ACPAs; this mechanism is called “molecular mimicry”.^3^ The two most implicated *P. gingivalis* antigens are enolase and heat shock protein (HSP). Enolase from *P. gingivalis* shares 51.4% amino acid identity with its human ortholog, α-enolase.^113^ Its citrullination in periodontal tissue is thought to lead to the generation of antibodies that also recognize citrullinated human α-enolase peptide 1 (anti-CEP1 antibodies).^113^ In a DR4-IE–transgenic mouse arthritis model, immunization with bacterial enolase induced synovial hyperplasia and bone erosion associated with induction of anti-CEP1 antibodies.^114^ HSP60 from *P. gingivalis* also contains a peptide epitope that is recognized by antibodies in the serum of RA patients.^115^ Many other bacteria of the oral biofilm express enolase and HSP60, which are highly conserved and thus may also contribute to molecular mimicry and disruption of immune tolerance in inflamed gingival mucosa.^3^

The generation of NETs by neutrophils in periodontitis led to the generation of neoepitopes as in synovial tissue.^99^ In periodontitis patients, the increased production of NETs suggests that they are generated during chronic gingival infection and may be a source of self-antigens, which could lead to RA.^116^

#### Danger-associated molecular patterns

During chronic inflammation, failure to clear apoptotic cells leads to secondary necrosis and release of cellular contents, including intracellular danger-associated molecular patterns (DAMPs), which accelerate local and systemic inflammation.^3^ Thus, DAMPs can disrupt tolerance to self-antigens and contribute to autoimmune diseases such as RA.^3^ Alarmins (HSP, hyaluronan, uric acid, high-mobility group box protein 1 (HMGB1), S100 proteins, IL-1α, and IL-33) contribute to a group of DAMPs identified in the development of RA,^117^ and some of them, such as IL-33, are also potentially involved in periodontitis.^118^

The immunopathogenic similarities between RA and periodontitis, their possible common etiology and the osteoclastogenic role of periodontopathogenic bacteria raise questions about the development and/or circulation of common OCPs in the two diseases. It has been demonstrated that OCPs under inflammatory conditions are not only more numerous but also different from those present under homeostatic conditions. The following section will develop the current knowledge on these precursors.

## Part II. Diversity of osteoclast precursors in chronic inflammatory diseases

### Osteoclastogenesis

Inflammatory bone loss is the hallmark of RA and periodontitis. In these diseases, the physiological balance between bone formation by osteoblasts and bone resorption by OCs is disrupted in favor of bone resorption (uncoupling).^10^ Unlike OCs, which are tightly attached to the bone matrix, OCPs are present in the BM and peripheral reservoirs (blood, spleen).^101^ Their differentiation into OCs is controlled by the RANK/RANKL/osteoprotegerin (OPG) molecular triad.^10^ Binding of RANKL to its receptor RANK on the surface of OCPs causes the activation of multiple signaling cascades and transcription factors,^119^ leading to OC differentiation and activation.^32^ OPG is a soluble decoy receptor that binds to RANKL and thus prevents RANKL/RANK interaction and osteoclastogenesis. The RANKL/OPG ratio is therefore crucial for bone resorption.^10^ Recently, a new pathway for the differentiation of PBMCs into OCs has been described in vivo: TGFβ priming reprograms the human MP response toward osteoclastogenesis and then allows TNF to induce OC differentiation independently of RANKL.^120^

**Fig. 3:**
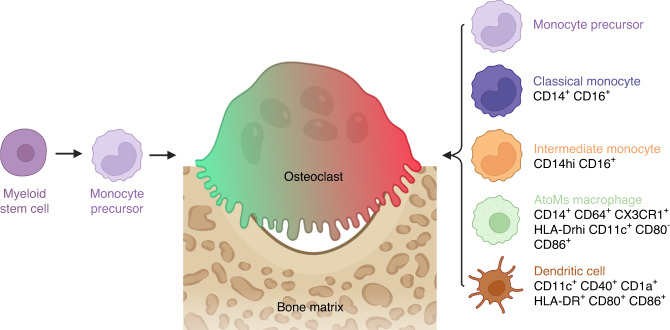
Origins of osteoclasts in physiological condition and pathological inflammatory bone resorption. In physiological conditions in adults, osteoclasts (OCs) involved in bone remodeling are derived from monocyte precursor cells. In pathological inflammatory bone resorption, OCs can also be derived from other precursors, which are classical or intermediate monocytes, inflammatory dendritic cells, or AtoMs. Major markers for these OCs are presented

**Fig. 4:**
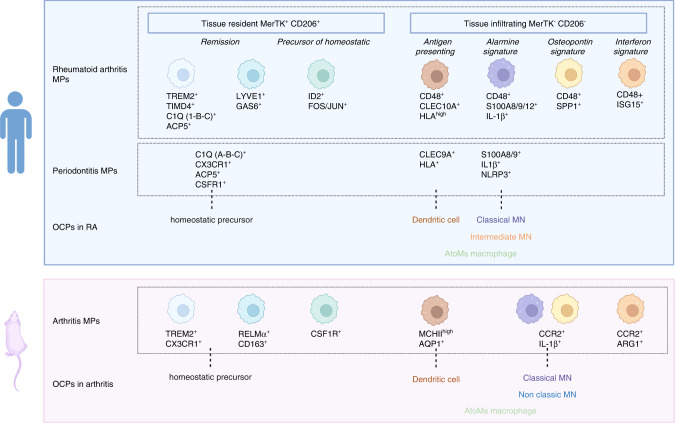
Relationships between macrophage clusters in arthritis, periodontitis and osteoclastic precursors. Different macrophagic clusters found in rheumatoid arthritis (Alivernini et al.^163^), periodontitis (Chen et al.^164^) in humans and in arthritic mice (Culemann et al.^165^) by single cell transcriptomics and the possible link between these clusters and osteoclastic precursors described in humans or mice upon homeostatic or inflammatory conditions

In steady states, OCs arise from the differentiation of the common MN/MP/OC progenitor (CD27^low^ B220^-^ CD11b^low/−^ c-Kit^+^ c-Fms^+^ Flt3^−^) present in BM.^121^ However, other OCPs have been described, particularly in the context of inflammation (Fig. 3, Tables 2 and 3). Figure 4 shows the relationships between the OCPs described below, the MPs of the gingival tissue in periodontitis patients, and the MPs of the synovium in RA patients.

**Table 2:** Phenotypes of osteoclast precursors described in mouse models of inflammatory diseases

Osteoclast precursor	Mouse model of inflammatory disease	Demonstrated	Precursor phenotype	References
Myeloid cells	SKG arthritis	In vitro, in vivo	CD11b^-/low^ Ly6C^high^ CD117^+^ CX3CR1^+^	^101,122^
Classic monocytes	Chronic inflammatory vaccine-based	In vivo	Lin^-^ Ly6C^hi^ CD11b^hi^	^124^
	CIA	In vitro, in vivo	CD11b^+^ CD115^+^ Ly6C^hi^	^101,127^
	Calvaria injection of *Pg*	In vitro, in vivo	CD11b^+^ CD115^+^ Ly6C^hi^	^119^
Nonclassic monocytes	hTNFtg and K/Bxn serum transfer arthritis	In vitro	CD11b^+^ CD115^+^ Ly6C^−^	^128^
Dendritic cells	IBD induced by transfer of naive CD4^+^ T cells into *Rag1*^*-/-*^ mice)	In vitro	CD11c^+^	^11^
	Coculture DC + CD4^+^ T cells + periodontopathogen antigen Osteopetrotic *oc/oc* mice	In vitro In vivo	CD11c^+^ CD11b^-^ F4/80^-^ Ly-6C^-^ CD31^-^ CD11c + MHC-II +	^137^ ^124^
AtoMs	CIA	In vitro, in vivo	CX3CR1^hi^ Ly6C^int^ F4/80^+^ I-A^+^/I-E^+^.	^16^

*CIA* collagen-induced arthritis, *Pg* Porphyromonas gingivalis, *IBD* inflammatory bowel disease, *DC* dendritic cells, *AtoMs* arthritis-associated osteoclastogenic macrophages

**Table 3:** Phenotypes of osteoclast precursors described in human diseases

Osteoclast precursor	Human disease	Demonstrated	Precursor phenotype	Reference
Classical monocytes	RA	In vitro, in vivo	CD14^+^ CD16^-^ Tyro3TK^+^	^14^
	RA	In vitro	CD14^+^ CD16^-^ Lin^−^ HLA-DR^+^	^131^
Intermediate monocytes	Psoriatic arthritis	In vitro, in vivo	CD14^high^ CD16^**+**^	^129^
	Periodontitis	In vivo	CD14 ^high^ CD16^**+**^ HLA-DR, PDL1, T2DM et CD47	^130^
Conventional predendritic cells	RA	In vitro	Lin^−^ HLA-DR^+^ CD14^−^ CD16^−^ CD11c^+^	^131^
Immature dendritic cells	RA	In vitro	CD1a^+^ CD14^-^ HLA-DR^int^ CD83low CD80low CD86low TRAP^+^	^15^
	Multiple myeloma	In vitro	Lyn-/CD11c + /Cd1c^+^/BDCA-3^+^/ HLA-DR^+^/CD80^-^	^142^
AtoMs	RA	In vivo	CX3CR1^+^ HLA-DR^hi^ CD11c^+^ CD80^−^ CD86^+^	^16^

*RA* rheumatoid arthritis, *AtoMs* arthritis-associated osteoclastogenic macrophages

### Monocyte-derived inflammatory osteoclast precursors

MNs are ubiquitous innate immune cells characterized by a high level of plasticity. Depending on environmental signals, they can differentiate into DCs, MPs, or OCs.^12^

In mouse models of inflammatory diseases detailed below, three subpopulations of MN-derived OCPs have been identified: myeloid cells (CD11b^–/lo^ Ly6C^hi^),^122^ classical MNs (or inflammatory MNs Ly6C^high^ CD11b^+^ Csf1r^+^ CCR2^high^ Cx3cr1^low^ Sell^+^),^123^ and nonclassical MNs (or patrolling MNs, Ly6C^low^ CD11b^+^ Csf1r^+^ CCR2^low^ CX3CR1^high^ Sell^-^).^123^

Myeloid cells from the BM, as in physiological conditions, can differentiate into OCs in SKG mice that spontaneously develop inflammatory arthritis.^122^ In vitro, characterization of these OCP progenitors identified a main subpopulation (80% of them) expressing the fractalkine receptor CX3CR1 that was highly enriched in OCPs^122^ (Table 2). However, a chronic inflammatory vaccine-based murine model (repetitive vaccination with heat-killed *Mycobacterium tuberculosis* H37 Ra (BCG)) demonstrated the existence of a homeostatic OCP (Lin^-^ Ly6C^hi^CD11b^lo^) population that was predominant in the BM of noninflamed control mice and was unresponsive in inflamed mice.^124^

Under inflammatory conditions, classical MNs migrate more rapidly and in greater numbers from the BM and spleen to inflammatory sites than nonclassical MNs.^12,125^ In vitro treatment of BM cells with TNF-α demonstrated that classical MNs were much more efficient than nonclassical MNs in differentiating into mature OCs.^126^ The chronic inflammatory vaccine-based murine model described above also demonstrated the existence of inflammatory OCPs (Lin^-^ Ly6C^hi^ CD11b^hi^), which represented a minor population in noninflamed control mice, but they expanded significantly in the BM and blood in inflamed mice.^124^ This population showed an immune-inflammatory proteome, T suppressive activity, and resorption potential after differentiation into OCs that was higher than that of homeostatic OCPs (Lin^-^ Ly6C^hi^CD11b^lo^)^124^ (Table 2). In the CIA murine model, Ly6C^high^ blood monocytosis was associated with arthritis, and classical MNs migrated more specifically to inflamed joints, contributing to bone erosion by differentiating into OCs.^127^ Finally, in a mouse calvarial infection model with repetitive injections of live *P. gingivalis* into the subcutaneous tissue of the parietal bone, the number of classical MNs increased significantly in the BM and spleen compared to control mice.^119^ These MNs showed both proinflammatory and pro-osteoclastogenic potential in vivo as well as in vitro^119^ (Table 2). They also overexpressed the Foxm1 gene, which is a transcription factor that plays a critical role in regulating the osteoclastic potential of OCPs in arthritis.^101^

Nonclassical MNs are thought to be involved in joint bone destruction in hTNFtg and serum K/BxN transfer models of arthritis.^128^ Their numbers in the blood and spleen correlate positively with markers of joint destruction, and their osteoclastic potential in vitro is greater than that of classical MNs.^128^

In humans, three subpopulations of MN-derived OCPs have been described: classical MNs (CD14^+^ CD16^−^),^14^ intermediate MNs (CD14^high^ CD16^+^),^129,130^ and a circulating myeloid population (CD14^−^ CD16^−^ CD11c^+^).^131^

Classical MNs are OCPs in healthy individuals and RA patients, since their osteoclastic potential has been confirmed in vitro.^14^ RA patients showed increased expression of the Tyro3TK receptor compared to intermediate MNs, which promoted differentiation into OCs. The levels of Tyro3TK^+^ classical MNs were positively correlated with the DAS28 activity score (DAS28-ESR) and serum IgM levels^14^ (Table 3).

Intermediate MNs responded to IL-17A by forming larger OCs with a higher resorptive capacity than in the absence of this cytokine.^132^ Analysis of peripheral blood from patients with psoriatic arthritis showed a significant increase in intermediate MNs compared with healthy controls.^129^ In vitro, these intermediate MNs differentiated into OCs, and their level of CD16 expression was positively correlated with the extent of bone resorption^129^ (Table 3). Therefore, they could have been in a transitional state toward differentiation into OCs. This significantly higher proportion of intermediate MNs has also been observed in gingival tissue samples from periodontitis patients compared to healthy patients.^130^ These MNs overexpressed HLA-DR, CD274, T2DM, and CD47, suggesting an inflammatory state^130^ (Table 3). However, analysis of peripheral blood from RA patients before the initiation of treatment showed that proportions of intermediate MNs were not significantly higher than in healthy patients.^133^ Although there was a significant positive correlation between the frequency of intermediate MNs and Th17 CXCR3^+^ cells in RA patients, neither intermediate MNs nor Th17 CXCR3^+^ cells were associated with bone density or bone microarchitecture parameters in RA patients.^133^

The circulating CD14^−^ CD16^−^ CD11c^+^ myeloid population rapidly differentiated into more numerous and larger OCs than those differentiated from CD14^+^ OCPs.^131^ It was reported that 56% of patients with moderate-to-severe active RA were unresponsive to TNF-α treatment because TNF-α inhibited osteoclastic differentiation of classical or intermediate MN OCPs (CD14^+^) but not myeloid OCPs (CD11c^+^).^131^

### Dendritic cell-derived inflammatory osteoclast precursors

DCs are sentinel cells of the immune system that have the ability to migrate to lymphoid organs, where they present antigens and thus activate naive T cells.^12^ Initially, DCs were considered to be fully differentiated cells.^12^ However, in recent years, it has been shown that immature DCs can differentiate into OCs.^12^ DCs, therefore, exhibit plasticity in response to their environment.^12^ As described above for MNs, the differentiation of DCs into OCs has been reported both in vitro and in vivo in a number of inflammatory diseases (such as inflammatory bowel disease or RA) but never in a healthy context.^12,134^

In mice, in infectious or inflammatory conditions, inflammatory DCs are generated from classical Ly6C^high^ MNs that are recruited to tissues, resulting in T-cell activation in draining lymph nodes.^12^ In addition to classical DC markers (CD11c, MHC-II, CD11b), these inflammatory DCs also express Mrc1, F4/80, Csf1r, Fcgr1, and FcƐRI. The latter two markers enable the distinction of inflammatory DCs from MPs.^134^ However, the differentiation of cDCs into OCs requires the presence of inflammatory CD4^+^ T cells and high levels of RANKL.^135^ In vitro, TNF-α and IL-1β positively regulated the differentiation of murine immature cDCs into OCs, whereas IFN-α inhibited this process.^136^ OCs differentiated from BM-derived DCs were shown to efficiently stimulate TN-Fα-producing CD4^+^ T cells in an antigen-dependent manner and were thus referred to as “inflammatory OCs” (iOCs), whereas those derived from BM-MNs stimulate FoxP3^+^ CD4^+^ regulatory T cells.^11^ OCs generated from inflammatory bowel disease mice have equivalent inflammatory capacity, while OCs obtained from healthy mice stimulate Treg cells.^11^ Interestingly, Cx3cr1 was the first marker identified for DC-derived OCs but was expressed in only approximately 20% of these cells. A comparison of Cx3cr1^+^ and Cx3cr1^−^ DC-derived OCs revealed that only the Cx3cr1^−^ DC-derived OCs had a higher matrix dissolution activity and a greater ability to induce T-cell proliferation. In addition, Cx3cr1^+^ iOCs expressed immunosuppressive factors and controlled the immune function of Cx3cr1^−^ iOCs, revealing heterogeneity in OC populations.^13^ Of note, IL-17A has been shown to be a key inducer of iOC formation.^11,135^ Several studies have looked for links between DCs and alveolar bone loss associated with periodontitis. In vitro, the interaction of murine CD11c^+^ DCs with T cells in the presence of antigens from bacteria involved in periodontitis was sufficient to give rise to DC-derived OCs^137^ (Table 2).

In vitro, OCs differentiated from human MN-derived DCs show the same bone resorption capacity as those differentiated from MNs, but they differentiate more rapidly and form OCs with more nuclei.^15^ The presence of CD1a^+^ DCs has been described in the synovium of RA patients.^138,139^ CD1a^+^ DCs (RANK^+^ CD14- HLA a,b,c^int^, HLA DR^int^, CD80^low^, CD83^low^, and CD86^low^) were closer to OCs than MNs.^138^ They express a higher level of essential osteoclastogenic genes *(RANK, c-Fms, TREM-2, BLNK*, and *TRAP)* than MNs, which gives them a greater ability to differentiate into iOCs.^138^ In vitro, the differentiation of CD1a^+^ CD14^−^ DCs from the blood of healthy adults into OCs is faster and more efficient than MN-derived OC formation.^15^ Recombinant human TNF-α, IL-1α, IL-17A, or synovial fluid from RA patients enhances the differentiation of these DCs^15^ (Table 3). Blood cell culture from patients with Crohn’s disease showed that Th17 cells induced the differentiation of MNs and DCs into OCs,^140^ reinforcing the role of IL-17A as a key inducer of OC formation in an inflammatory context.^11^ In RA patients, inflammation and severity of bone erosion have been correlated with the presence of Th17 cells in the blood or joints,^141^ a condition associated in mice with the emergence of DC-derived OCs.^135^ Furthermore, BM DCs from patients with multiple myeloma overexpressed the IL-17 receptor and differentiated into functional OCs after stimulation with IL-17A and CSF1 in vitro^142^ (Table 3). In the gingiva of periodontitis patients, the density of immature CD1a^+^ DCs was higher in individuals with a strong inflammatory cell infiltrate than in those with a mild infiltrate.^143^ The increase in the number of immature DCs was associated with the initial stage of periodontal disease.^143^ In addition, Th17 cells and RANKL have been detected in inflamed gingival cells.^144,145^ However, to date, the presence of gingival OCs from DCs has not been evidenced in periodontitis.

### Macrophage-derived inflammatory osteoclast precursors

The development of high-throughput technologies, such as single-cell RNA sequencing and spatial transcriptomics, has enabled progress in the characterization of cell subpopulations. MPs have a wide range of tissue-specific functions in homeostatic and pathological conditions.^146^ In response to stimuli from the local microenvironment, MPs may exhibit a wide spectrum of phenotypes.^146^ More than 10 years ago, two major phenotypes of MN-derived infiltrating MPs were described: classically activated M1 or alternatively activated M2 MPs.^147^ Recently, new subpopulations have been described according to their activation status, origins, and homeostatic/pathological functions.^146^ In RA, MPs play a key role in the initiation and chronicity of the disease, interacting with fibroblast-like synoviocytes and innate and adaptive immune cells, leading to the progression of synovitis and bone erosion.^146^ Hasegawa et al. identified arthritis-associated osteoclastogenic macrophages (AtoMs), which are bone marrow-derived OCPs with a high ability to differentiate into OCs in the CIA murine model (CX3CR1^hi^ Ly6C^int^ F4/80^+^ I-A^+^/I-E^+^) (Table 2) and in RA patients (CX3CR1^+^ HLA-DR^hi^ CD11c^+^ CD80^−^ CD86^+^)^16^ (Table 3). In the CIA murine model, 10% of AtoMs from the pannus (i.e., inflamed synovium) of mice differentiated into mature OCs.^148^ Intravital imaging analysis of the pannus–bone interface confirmed that some AtoMs were OCPs transitioning into mature OCs. These OCs actively resorbed the bone matrix without migrating to the bone surface,^148^ unlike physiological BM OCs, which are in close contact with osteoblasts and migrate slowly to the bone surface.^149^ In addition, therapy targeting AtoMs (thiostrepton, an inhibitor of FOXM1 activity) resulted in a decrease in bone erosion in CIA mice without affecting homeostatic bone remodeling in healthy mice, both in vitro and in vivo.^16^ AtoMs had the structural phenotype of MPs; however, they also expressed CD11c, HLA-DR, and CD80/CD86.^150^ Thus, they shared functional characteristics with MPs and DCs, unlike OCPs present in BM and blood under physiological conditions, which do not express HLA-DR.^150^ These results imply that the actors in resorption differ between homeostatic bone remodeling and inflammatory bone resorption.^151^

All the reviewed data suggest that in chronic inflammatory diseases, OCs can originate from different OCPs (MNs, DCs, or MPs) that are not necessarily described in homeostatic conditions (Fig. 3).^12^ Given the phenotypic proximity of the different OCPs, it is questionable whether they originate from different cell types. Furthermore, intravital imaging of OCs in mouse tibia has shown that mature OCs stimulated by soluble RANKL were able to fuse but also to fission into smaller daughter cells called “osteomorphs”, themselves able to merge with each other or with large OCs.^152,153^ This process of cell recycling is not observed in a steady state and is inhibited by OPG treatment.^152^ Thus, depending on the cells present in the vicinity, it is very likely that OCs are formed by a mixture of different OCPs rather than being derived from pure OCPs, reflecting a high degree of flexibility and plasticity depending on the pathological conditions.^12^ The inflammatory environment, therefore, plays a key pathogenic role.

## Part III. Therapeutic targeting of osteoclast precursors in rheumatoid arthritis and periodontitis

In recent decades, our understanding of immunopathological mechanisms has led to the development of treatments targeting specific components of the immune response. The emergence of the concept of OC subsets specifically involved in inflammation and associated with pathological and disabling bone resorption provides new therapeutic opportunities for RA or periodontitis. Indeed, therapies targeted at OCs with inflammatory potential would only focus on inflammatory bone resorption while preserving homeostatic bone remodeling. However, a full characterization of such OCs, including the identification of their progenitors and their mechanisms of differentiation, is still needed to enable the development of such targeted therapies.

Therapeutic anti-inflammatory approaches targeting, in particular, the MN/MP OCPs that are promising in RA could be of potential interest in periodontitis due to the immunopathogenic similarities between the two diseases. Currently, only two therapies targeting OCPs described in RA have shown promising results. These are cytotoxic T lymphocyte antigen-4-immunoglobulin (CTLA-4-Ig) and thiostrepton, which affect monocytic cells described to participate in osteoclastogenesis in RA.^16,154^

CTLA-4-Ig (Abatacept) is a genetically engineered biological agent that, similar to the immunoregulatory receptor CTLA-4, binds to CD80 and CD86 on antigen-presenting cells, thereby preventing binding to T cells.^155^ It has been shown to protect against joint destruction in RA.^156^ In vitro, CTLA-4-Ig directly affects the phenotype and function of MNs and MPs from RA patients.^157^ Additionally, in vitro, CTLA-4-Ig inhibited the differentiation of MNs into OCs in a dose-dependent manner.^158^ Pretreatment of these cells with TNF-α to mimic an inflammatory condition enhanced the potential of CTLA-4-Ig to inhibit osteoclastogenesis.^158^ Intravital synovial imaging of mice with CIA revealed that CTLA-4-Ig binds to CX3CR1^lo/+^ Ly6C^int^ CD80^+^ CD86^+^ MPs.^151^ Thus, this binding could inhibit osteoclast differentiation, resulting in bone protective effects.

Thiostrepton is an antibiotic that is also an inhibitor of FoxM1, a transcriptional activator involved in cell proliferation.^159^ FoxM1 plays an important role in carcinogenesis, and the invasive phenotype of a tumor is analogous to the pannus in arthritis, which erodes the bone surface and destroys joints.^159^ FoxM1 also promotes synovial fibroblast growth in RA through activation of the Wnt/β-catenin pathway,^160^ where it acts as a regulator of inflammation and chondrocyte cell death via JAK1/STAT3.^161^ Hasegawa et al. demonstrated that FoxM1 was the transcription factor responsible for regulating the osteoclastogenic potential of AtoM OCPs.^16^ In the CIA model, inhibition of FoxM1 by thiostrepton injection attenuated arthritis scores, inflammatory cytokine expression in the synovium, and joint bone destruction. In vitro, thiostrepton significantly inhibited RANKL-induced osteoclastogenesis of human AtoMs.^16^

Although promising results have been reported for CTLA-4-Ig and thiostrepton in RA, no treatments targeting specific OCPs have been studied in periodontitis due to the lack of data regarding OCPs in this disease. However, given the immunopathogenic similarities between RA and periodontitis, these therapies could be interesting to explore once OCPs are characterized.

Another promising therapeutic avenue could be miRNAs. Recently, it has been shown that microRNAs are involved in the regulation of osteoclastogenesis and in particular, in the formation and maturation of OCPs and OCs.^162^ In a CIA murine model, the in vivo delivery of the microRNA miR-146a targeting Ly6C^high^ MNs reduced OC differentiation and prevented pathological bone destruction.^127^

## Conclusion

This review highlighted many immunopathogenic similarities between RA and periodontitis. The hypothesis of a common etiology for both diseases could be an explanatory factor for these similarities and reinforces the relevance in finding common targeted therapies. The fact that OCs are involved both in inflammation and in associated bone resorption in the two pathologies is certainly of importance. Therefore, new therapeutic approaches that specifically target such Ocs or their precursors without altering homeostatic bone remodeling would indeed be promising. Future investigations are required to clearly identify OCPs responsible for the emergence of Ocs with an inflammatory role in RA and periodontitis, to decipher their mechanisms of differentiation and to determine whether they could be considered a major target for therapeutic strategies in patients with RA and/or periodontitis. Recent data suggest that under inflammatory conditions, Ocs are not only osteoclastic cells but also immune cells involved in inflammation and regulatory processes. The development of high-throughput technologies (scRNA-seq, spatial transcriptomics, imaging mass spectrometry, etc.) will allow us to better characterize the osteoclastic cell subpopulations and understand their interactions in the osteoimmunology microenvironment. These steps are essential for developing treatments that target bone resorption without deregulating homeostatic conditions. New antiresorptive agents targeting specific OCPs would be indicated for periodontitis patients who respond poorly to mechanical treatment or who relapse rapidly to stabilize the disease and prevent its local and systemic effects.

## References

[1] Silman AJ, Pearson JE (2002). Epidemiology and genetics of rheumatoid arthritis. Arthritis Res..

[2] Mikuls TR, Payne JB, Deane KD, Thiele GM (2016). Autoimmunity of the lung and oral mucosa in a multisystem inflammatory disease: The spark that lights the fire in rheumatoid arthritis?. J. Allergy Clin. Immunol..

[3] Potempa J, Mydel P, Koziel J (2017). The case for periodontitis in the pathogenesis of rheumatoid arthritis. Nat. Rev. Rheumatol..

[4] Papapanou PN (2018). Periodontitis: consensus report of workgroup 2 of the 2017 World Workshop on the Classification of Periodontal and Peri-Implant Diseases and Conditions. J. Clin. Periodontol..

[5] Eke PI (2018). Periodontitis in US adults: National Health and Nutrition Examination Survey 2009–2014. J. Am. Dent. Assoc..

[6] Kassebaum NJ (2014). Global burden of severe periodontitis in 1990–2010: a systematic review and meta-regression. J. Dent. Res..

[7] Costa FO (2015). Surgical and non-surgical procedures associated with recurrence of periodontitis in periodontal maintenance therapy: 5-year prospective study. PLoS One.

[8] Socransky SS, Haffajee AD (2005). Periodontal microbial ecology. Periodontol.

[9] Di Benedetto A, Gigante I, Colucci S, Grano M (2013). Periodontal disease: linking the primary inflammation to bone loss. Clin. Dev. Immunol..

[10] Boyle WJ, Simonet WS, Lacey DL (2003). Osteoclast differentiation and activation. Nature.

[11] Ibáñez L (2016). Inflammatory osteoclasts prime TNFα-Producing CD4+ T cells and express CX3CR1. J. Bone Miner. Res..

[12] Madel MB (2019). Immune function and diversity of osteoclasts in normal and pathological conditions. Front. Immunol..

[13] Madel MB (2020). Dissecting the phenotypic and functional heterogeneity of mouse inflammatory osteoclasts by the expression of cx3cr1. Elife.

[14] Xue J (2020). CD14+CD16-monocytes are the main precursors of osteoclasts in rheumatoid arthritis via expressing Tyro3TK. Arthritis Res. Ther..

[15] Rivollier A (2004). Immature dendritic cell transdifferentiation into osteoclasts: a novel pathway sustained by the rheumatoid arthritis microenvironment. Blood.

[16] Hasegawa T (2019). Identification of a novel arthritis-associated osteoclast precursor macrophage regulated by FoxM1. Nat. Immunol..

[17] Marotte H (2006). The association between periodontal disease and joint destruction in rheumatoid arthritis extends the link between the HLA‐DR shared epitope and severity of bone destruction. Ann. Rheum. Dis..

[18] Stein J, Reichert S, Gautsch A, Machulla HKG (2003). Are there HLA combinations typical supporting for or making resistant against aggressive and/or chronic periodontitis?. J. Periodontal. Res..

[19] Qiao Y (2020). Rheumatoid arthritis risk in periodontitis patients: a systematic review and meta-analysis. Joint Bone Spine.

[20] Marchesan JT (2013). Porphyromonas gingivalis oral infection exacerbates the development and severity of collagen-induced arthritis. Arthritis Res. Ther..

[21] Corrêa MG (2017). Periodontitis increases rheumatic factor serum levels and citrullinated proteins in gingival tissues and alter cytokine balance in arthritic rats. PLoS One.

[22] Lübcke PM (2019). Periodontal treatment prevents arthritis in mice and methotrexate ameliorates periodontal bone loss. Sci. Rep..

[23] Courbon G (2019). Porphyromonas gingivalis experimentally induces periodontis and an anti-CCP2-associated arthritis in the rat. Ann. Rheum. Dis..

[24] Li Y (2022). The relationship between porphyromonas gingivalis and rheumatoid arthritis: a meta-analysis. Front. Cell. Infect. Microbiol..

[25] Berthelot JM (2022). Another look at the contribution of oral microbiota to the pathogenesis of rheumatoid arthritis: a narrative review. Microorganisms.

[26] Moura MF (2021). Nonsurgical periodontal therapy decreases the severity of rheumatoid arthritis and the plasmatic and salivary levels of RANKL and Survivin: a short-term clinical study. Clin. Oral. Investig..

[27] Oliveira SR (2022). Are neutrophil extracellular traps the link for the cross-talk between periodontitis and rheumatoid arthritis physiopathology?. Rheumatology.

[28] González-Febles J, Sanz M (2000). Periodontitis and rheumatoid arthritis: what have we learned about their connection and their treatment?. Periodontology.

[29] Perricone C (2019). Porphyromonas gingivalis and rheumatoid arthritis. Curr. Opin. Rheumatol..

[30] Hashimoto H, Hashimoto S, Shimazaki Y (2022). Functional impairment and periodontitis in rheumatoid arthritis. Int. Dent. J..

[31] Rodríguez-Lozano B (2019). Association between severity of periodontitis and clinical activity in rheumatoid arthritis patients: a case-control study. Arthritis Res. Ther..

[32] Lin YJ, Anzaghe M, Schülke S (2020). Update on the pathomechanism, diagnosis, and treatment options for rheumatoid arthritis. Cells.

[33] Sandros J (2000). Cytokine responses of oral epithelial cells to Porphyromonas gingivalis infection. J. Dent. Res..

[34] Usui M (2016). Gingival epithelial cells support osteoclastogenesis by producing receptor activator of nuclear factor kappa B ligand via protein kinase A signaling. J. Periodontal Res..

[35] Jang JY, Song IS, Baek KJ, Choi Y, Ji S (2017). Immunologic characteristics of human gingival fibroblasts in response to oral bacteria. J. Periodontal. Res..

[36] Belibasakis GN (2007). Regulation of RANKL and OPG gene expression in human gingival fibroblasts and periodontal ligament cells by Porphyromonas gingivalis: a putative role of the Arg-gingipains. Microb. Pathog..

[37] Lefèvre S (2009). Synovial fibroblasts spread rheumatoid arthritis to unaffected joints. Nat. Med..

[38] Lewis MJ (2019). Molecular portraits of early rheumatoid arthritis identify clinical and treatment response phenotypes. Cell Rep..

[39] Sharawi H (2021). The prevalence of gingival dendritic cell subsets in periodontal patients. J. Dent. Res..

[40] Kinane DF, Stathopoulou PG, Papapanou PN (2017). Periodontal diseases. Nat. Rev. Dis. Prim..

[41] Mantovani A, Cassatella MA, Costantini C, Jaillon S (2011). Neutrophils in the activation and regulation of innate and adaptive immunity. Nat. Rev. Immunol..

[42] Hajishengallis G (2015). Periodontitis: from microbial immune subversion to systemic inflammation. Nat. Rev. Immunol..

[43] Hajishengallis G, Chavakis T (2021). Local and systemic mechanisms linking periodontal disease and inflammatory comorbidities. Nat. Rev. Immunol..

[44] Ling MR, Chapple IL, Matthews JB (2015). Peripheral blood neutrophil cytokine hyper-reactivity in chronic periodontitis. Innate Immun..

[45] Coutant F, Miossec P (2016). Altered dendritic cell functions in autoimmune diseases: distinct and overlapping profiles. Nat. Rev. Rheumatol..

[46] Lebre MC (2008). Rheumatoid arthritis synovium contains two subsets of CD83−DC-LAMP− dendritic cells with distinct cytokine profiles. Am. J. Pathol..

[47] Jongbloed SL (2006). Enumeration and phenotypical analysis of distinct dendritic cell subsets in psoriatic arthritis and rheumatoid arthritis. Arthritis Res. Ther..

[48] Segura E (2013). Human inflammatory dendritic cells induce Th17 cell differentiation. Immunity.

[49] Page G, Miossec P (2004). Paired synovium and lymph nodes from rheumatoid arthritis patients differ in dendritic cell and chemokine expression. J. Pathol..

[50] Kurgan S, Kantarci A (2018). Molecular basis for immunohistochemical and inflammatory changes during progression of gingivitis to periodontitis. Periodontol 2000.

[51] Rankin L, Groom J, Mielke LA, Seillet C, Belz GT (2013). Diversity, function, and transcriptional regulation of gut innate lymphocytes. Front. Immunol..

[52] Chemin K, Gerstner C, Malmström V (2019). Effector functions of CD4+ T cells at the site of local autoimmune inflammation-lessons from rheumatoid arthritis. Front. Immunol..

[53] Gemmell E, Seymour GJ (2004). Immunoregulatory control of Th1/Th2 cytokine profiles in periodontal disease. Periodontol 2000.

[54] Liao C, Zhang C, Yang Y (2017). Pivotal roles of interleukin-17 as the epicenter of bone loss diseases. Curr. Pharm. Des..

[55] de Molon RS, Rossa C, Thurlings RM, Cirelli JA, Koenders MI (2019). Linkage of periodontitis and rheumatoid arthritis: current evidence and potential biological interactions. Int. J. Mol. Sci..

[56] Cascão R (2010). Identification of a cytokine network sustaining neutrophil and Th17 activation in untreated early rheumatoid arthritis. Arthritis Res. Ther..

[57] Zhang Y, Li Y, Lv TT, Yin ZJ, Wang XB (2015). Elevated circulating Th17 and follicular helper CD4(+) T cells in patients with rheumatoid arthritis. APMIS.

[58] Garlet GP (2010). Regulatory T cells attenuate experimental periodontitis progression in mice. J. Clin. Periodontol..

[59] Aletaha D (2010). Rheumatoid arthritis classification criteria: an American College of Rheumatology/European League Against Rheumatism collaborative initiative. Arthritis Rheum..

[60] Gerlag DM (2019). Effects of B-cell directed therapy on the preclinical stage of rheumatoid arthritis: the PRAIRI study. Ann. Rheum. Dis..

[61] Ohlrich EJ, Cullinan MP, Seymour GJ (2009). The immunopathogenesis of periodontal disease. Aust. Dent. J..

[62] Wu F (2021). B cells in rheumatoid arthritis: pathogenic mechanisms and treatment prospects. Front. Immunol..

[63] Settem RP, Honma K, Chinthamani S, Kawai T, Sharma A (2021). B-Cell RANKL contributes to pathogen-induced alveolar bone loss in an experimental periodontitis mouse model. Front. Physiol..

[64] Hatipoğlu M (2022). B cell depletion in patients with rheumatoid arthritis is associated with reduced IL-1β in GCF. Clin. Oral. Investig..

[65] Wang Y (2017). B10 cells alleviate periodontal bone loss in experimental periodontitis. Infect. Immun..

[66] R L (2017). Rheumatoid arthritis and periodontal disease: what are the similarities and differences?. Int. J. Rheum. Dis..

[67] Lam J (2000). TNF-α induces osteoclastogenesis by direct stimulation of macrophages exposed to permissive levels of RANK ligand. J. Clin. Invest..

[68] Marahleh A (2019). TNF-α directly enhances osteocyte RANKL expression and promotes osteoclast formation. Front. Immunol..

[69] Brennan FM, McInnes IB (2008). Evidence that cytokines play a role in rheumatoid arthritis. J. Clin. Invest..

[70] Romero-Sanchez C (2017). Is the treatment with biological or non-biological DMARDS a modifier of periodontal condition in patients with rheumatoid arthritis. Curr. Rheumatol. Rev..

[71] Avci AB, Feist E, Burmester GR (2018). Targeting IL-6 or IL-6 receptor in rheumatoid arthritis: what’s the difference?. BioDrugs.

[72] Amarasekara DS (2018). Regulation of osteoclast differentiation by cytokine networks. Immune Netw..

[73] Fossiez F (1996). T cell interleukin-17 induces stromal cells to produce proinflammatory and hematopoietic cytokines. J. Exp. Med..

[74] Chabaud M (2000). Contribution of interleukin 17 to synovium matrix destruction in rheumatoid arthritis. Cytokine.

[75] Van Bezooijen RL, Papapoulos SE, Löwik CWGM (2001). Effect of interleukin-17 on nitric oxide production and osteoclastic bone resorption: is there dependency on nuclear factor-κB and receptor activator of nuclear factor κB (RANK)/RANK ligand signaling?. Bone.

[76] Adibrad M (2012). Signs of the presence of Th17 cells in chronic periodontal disease. J. Periodontal. Res..

[77] Chukkapalli S (2016). Periodontal bacterial colonization in synovial tissues exacerbates collagen-induced arthritis in B10.RIII mice. Arthritis Res. Ther..

[78] de Aquino SG (2017). The aggravation of arthritis by periodontitis is dependent of IL-17 receptor A activation. J. Clin. Periodontol..

[79] Kunwar S, Dahal K, Sharma S (2016). Anti-IL-17 therapy in treatment of rheumatoid arthritis: a systematic literature review and meta-analysis of randomized controlled trials. Rheumatol. Int..

[80] Schett G, Dayer JM, Manger B (2016). Interleukin-1 function and role in rheumatic disease. Nat. Rev. Rheumatol..

[81] Akitsu A (2015). IL-1 receptor antagonist-deficient mice develop autoimmune arthritis due to intrinsic activation of IL-17-producing CCR2(+)Vγ6(+)γδ T cells. Nat. Commun..

[82] Gaffen SL, Hajishengallis G (2008). A new inflammatory cytokine on the block: re-thinking periodontal disease and the Th1/Th2 paradigm in the context of Th17 cells and IL-17. J. Dent. Res..

[83] Singh JA (2010). Biologics for rheumatoid arthritis: an overview of Cochrane reviews. Sao Paulo Med. J..

[84] Ren B (2021). Anti-inflammatory effect of IL-1ra-loaded dextran/PLGA microspheres on Porphyromonas gingivalis lipopolysaccharide-stimulated macrophages in vitro and in vivo in a rat model of periodontitis. Biomed. Pharmacother..

[85] Pettit AR (2001). TRANCE/RANKL knockout mice are protected from bone erosion in a serum transfer model of arthritis. Am. J. Pathol..

[86] Cloitre A (2019). IL-36γ is a pivotal inflammatory player in periodontitis-associated bone loss. Sci. Rep..

[87] Kassem A (2015). Porphyromonas gingivalis stimulates bone resorption by enhancing RANKL (receptor activator of NF-κB ligand) through activation of toll-like receptor 2 in osteoblasts. J. Biol. Chem..

[88] Izati AF, Wong KK, Hussin C, Maraina C (2020). IL-23/IL-17 axis in the pathogenesis and treatment of systemic lupus erythematosus and rheumatoid arthritis. Malays. J. Pathol..

[89] Peichl P (2020). Abatacept retention and clinical outcomes in Austrian patients with rheumatoid arthritis: real-world data from the 2-year ACTION study. Wien. Med. Wochenschr..

[90] Mayer Y, Elimelech R, Balbir-Gurman A, Braun-Moscovici Y, Machtei EE (2013). Periodontal condition of patients with autoimmune diseases and the effect of anti-tumor necrosis factor-α therapy. J. Periodontol..

[91] Mayer Y, Balbir-Gurman A, Machtei EE (2009). Anti-tumor necrosis factor-alpha therapy and periodontal parameters in patients with rheumatoid arthritis. J. Periodontol..

[92] Schiefelbein R, Jentsch HFR (2018). Periodontal conditions during arthritis therapy with TNF-α blockers. J. Clin. Diagn. Res..

[93] Ancuța C (2021). Exploring the role of interleukin-6 receptor inhibitor tocilizumab in patients with active rheumatoid arthritis and periodontal disease. J. Clin. Med..

[94] Brianti P, Paolino G, Mercuri SR (2020). Successful use and safety of secukinumab in psoriatic patients with periodontitis: a valid therapeutic option. Dermatol. Ther..

[95] Coat J (2015). Anti-B lymphocyte immunotherapy is associated with improvement of periodontal status in subjects with rheumatoid arthritis. J. Clin. Periodontol..

[96] Rooney CM, Mankia K, Emery P (2020). The role of the microbiome in driving RA-related autoimmunity. Front. Cell Dev. Biol..

[97] Carrion J (2012). Microbial carriage state of peripheral blood dendritic cells (DCs) in chronic periodontitis influences DC differentiation, atherogenic potential. J. Immunol..

[98] Totaro MC (2013). Porphyromonas gingivalis and the pathogenesis of rheumatoid arthritis: analysis of various compartments including the synovial tissue. Arthritis Res. Ther..

[99] Corsiero E, Pratesi F, Prediletto E, Bombardieri M, Migliorini P (2016). NETosis as source of autoantigens in rheumatoid arthritis. Front. Immunol..

[100] Pratesi F (2014). Antibodies from patients with rheumatoid arthritis target citrullinated histone 4 contained in neutrophils extracellular traps. Ann. Rheum. Dis..

[101] Zhao Y (2020). Frontline science: characterization and regulation of osteoclast precursors following chronic Porphyromonas gingivalis infection. J. Leukoc. Biol..

[102] Zhang Y (2021). The association between periodontitis and inflammatory bowel disease: a systematic review and meta-analysis. Biomed. Res. Int..

[103] Rogier R (2017). Alteration of the intestinal microbiome characterizes preclinical inflammatory arthritis in mice and its modulation attenuates established arthritis. Sci. Rep..

[104] Sato K (2017). Aggravation of collagen-induced arthritis by orally administered Porphyromonas gingivalis through modulation of the gut microbiota and gut immune system. Sci. Rep..

[105] Mikuls TR (2014). Periodontitis and Porphyromonas gingivalis in patients with rheumatoid arthritis. Arthritis Rheumatol..

[106] Harvey GP (2013). Expression of peptidylarginine deiminase-2 and -4, citrullinated proteins and anti-citrullinated protein antibodies in human gingiva. J. Periodontal Res..

[107] Quirke AM (2014). Heightened immune response to autocitrullinated Porphyromonas gingivalis peptidylarginine deiminase: a potential mechanism for breaching immunologic tolerance in rheumatoid arthritis. Ann. Rheum. Dis..

[108] Laugisch O (2016). Citrullination in the periodontium-a possible link between periodontitis and rheumatoid arthritis. Clin. Oral. Investig..

[109] Sherina N (2022). Antibodies to a citrullinated Porphyromonas gingivalis epitope are increased in early rheumatoid arthritis, and can be produced by gingival tissue B cells: implications for a bacterial origin in RA etiology. Front. Immunol..

[110] Van den Steen PE (2002). Cleavage of denatured natural collagen type II by neutrophil gelatinase B reveals enzyme specificity, post-translational modifications in the substrate, and the formation of remnant epitopes in rheumatoid arthritis. FASEB J..

[111] Majeed ZN, Philip K, Alabsi AM, Pushparajan S, Swaminathan D (2016). Identification of gingival crevicular fluid sampling, analytical methods, and oral biomarkers for the diagnosis and monitoring of periodontal diseases: a systematic review. Dis. Markers.

[112] Guentsch A (2013). Cleavage of IgG1 in gingival crevicular fluid is associated with the presence of Porphyromonas gingivalis. J. Periodontal Res..

[113] Lundberg K (2008). Antibodies to citrullinated alpha-enolase peptide 1 are specific for rheumatoid arthritis and cross-react with bacterial enolase. Arthritis Rheum..

[114] Kinloch AJ (2011). Immunization with Porphyromonas gingivalis enolase induces autoimmunity to mammalian α-enolase and arthritis in DR4-IE-transgenic mice. Arthritis Rheum..

[115] Jeong E, Lee JY, Kim SJ, Choi J (2012). Predominant immunoreactivity of Porphyromonas gingivalis heat shock protein in autoimmune diseases. J. Periodontal Res..

[116] Hirschfeld J (2015). Neutrophil extracellular trap formation in supragingival biofilms. Int. J. Med. Microbiol..

[117] Nefla M, Holzinger D, Berenbaum F, Jacques C (2016). The danger from within: alarmins in arthritis. Nat. Rev. Rheumatol..

[118] Lapérine O (2016). Interleukin-33 and RANK-L interplay in the alveolar bone loss associated to periodontitis. PLoS One.

[119] Cai X (2020). Enhanced dual function of osteoclast precursors following calvarial Porphyromonas gingivalis infection. J. Periodontal Res..

[120] Xia Y (2022). TGFβ reprograms TNF stimulation of macrophages towards a non-canonical pathway driving inflammatory osteoclastogenesis. Nat. Commun..

[121] Xiao Y (2017). Macrophages and osteoclasts stem from a bipotent progenitor downstream of a macrophage/osteoclast/dendritic cell progenitor. Blood Adv..

[122] Charles JF (2012). Inflammatory arthritis increases mouse osteoclast precursors with myeloid suppressor function. J. Clin. Invest..

[123] Shi C, Pamer EG (2011). Monocyte recruitment during infection and inflammation. Nat. Rev. Immunol..

[124] Meirow Y (2022). Specific inflammatory osteoclast precursors induced during chronic inflammation give rise to highly active osteoclasts associated with inflammatory bone loss. Bone Res..

[125] Swirski FK (2009). Identification of splenic reservoir monocytes and their deployment to inflammatory sites. Science.

[126] Zhao Z (2015). TNF induction of NF-κB RelB enhances RANKL-induced osteoclastogenesis by promoting inflammatory macrophage differentiation but also limits it through suppression of NFATc1 expression. PLoS One.

[127] Ammari M (2018). Delivery of miR-146a to Ly6Chigh monocytes inhibits pathogenic bone erosion in inflammatory arthritis. Theranostics.

[128] Puchner A (2018). Non-classical monocytes as mediators of tissue destruction in arthritis. Ann. Rheum. Dis..

[129] Chiu YG (2010). CD16 (FcRγIII) as a potential marker of osteoclast precursors in psoriatic arthritis. Arthritis Res. Ther..

[130] Almubarak A, Tanagala KKK, Papapanou PN, Lalla E, Momen-Heravi F (2020). Disruption of monocyte and macrophage homeostasis in periodontitis. Front. Immunol..

[131] Ansalone C (2021). TNF is a homoeostatic regulator of distinct epigenetically primed human osteoclast precursors. Ann. Rheum. Dis..

[132] Sprangers S, Schoenmaker T, Cao Y, Everts V, de Vries TJ (2016). Different blood-borne human osteoclast precursors respond in distinct ways to IL-17A. J. Cell. Physiol..

[133] Drevinge C (2021). Intermediate monocytes correlate with CXCR3+ Th17 cells but not with bone characteristics in untreated early rheumatoid arthritis. PLoS One.

[134] Lapérine O, Blin-Wakkach C, Guicheux J, Beck-Cormier S, Lesclous P (2016). Dendritic-cell-derived osteoclasts: a new game changer in bone-resorption-associated diseases. Drug Discov. Today.

[135] Wakkach A (2008). Bone marrow microenvironment controls the in vivo differentiation of murine dendritic cells into osteoclasts. Blood.

[136] Speziani C (2007). Murine dendritic cell transdifferentiation into osteoclasts is differentially regulated by innate and adaptive cytokines. Eur. J. Immunol..

[137] Alnaeeli M, Penninger JM, Teng Y-TA (2006). Immune interactions with CD4 + T cells promote the development of functional osteoclasts from murine CD11c + dendritic cells. J. Immunol..

[138] Gallois A (2010). Genome-wide expression analyses establish dendritic cells as a new osteoclast precursor able to generate bone-resorbing cells more efficiently than monocytes. J. Bone Miner. Res..

[139] Page G, Miossec P (2005). RANK and RANKL expression as markers of dendritic cell-T cell interactions in paired samples of rheumatoid synovium and lymph nodes. Arthritis Rheum..

[140] Ciucci T (2015). Bone marrow Th17 TNFα cells induce osteoclast differentiation, and link bone destruction to IBD. Gut.

[141] Leipe J (2010). Role of Th17 cells in human autoimmune arthritis. Arthritis Rheum..

[142] Tucci M (2013). Immature dendritic cells in multiple myeloma are prone to osteoclast-like differentiation through interleukin-17A stimulation. Br. J. Haematol..

[143] Ribeiro Souto G, Queiroz CM, Nogueira Guimarães De Abreu MH, Oliveira Costa F, Alves Mesquita R (2014). Pro-inflammatory, Th1, Th2, Th17 cytokines and dendritic cells: a cross-sectional study in chronic periodontitis. PLoS One.

[144] Cardoso CR (2009). Evidence of the presence of T helper type 17 cells in chronic lesions of human periodontal disease. Oral. Microbiol. Immunol..

[145] Nagasawa T (2002). LPS-stimulated human gingival fibroblasts inhibit the differentiation of monocytes into osteoclasts through the production of osteoprotegerin. Clin. Exp. Immunol..

[146] Boutet MA (2021). Novel insights into macrophage diversity in rheumatoid arthritis synovium. Autoimmun. Rev..

[147] Mantovani A (2004). The chemokine system in diverse forms of macrophage activation and polarization. Trends Immunol..

[148] Hasegawa T (2021). Updating the pathophysiology of arthritic bone destruction: identifying and visualizing pathological osteoclasts in pannus. Immunol. Med..

[149] Furuya M (2018). Direct cell–cell contact between mature osteoblasts and osteoclasts dynamically controls their functions in vivo. Nat. Commun..

[150] Hasegawa T, Kikuta J, Ishii M (2021). Imaging of bone and joints in vivo: pathological osteoclastogenesis in arthritis. Int. Immunol..

[151] Hasegawa T (2020). Development of an intravital imaging system for the synovial tissue reveals the dynamics of CTLA-4 Ig in vivo. Sci. Rep..

[152] McDonald MM (2021). Osteoclasts recycle via osteomorphs during RANKL-stimulated bone resorption. Cell.

[153] Mabilleau G, Libouban H, Geoffroy V (2021). Osteomorphs as a tool for personalized medicine. Trends Endocrinol. Metab..

[154] Bozec A (2014). T cell costimulation molecules CD80/86 inhibit osteoclast differentiation by inducing the IDO/tryptophan pathway. Sci. Transl. Med..

[155] Bluestone JA, St. Clair EW, Turka LA (2006). CTLA4Ig: bridging the basic immunology with clinical application. Immunity.

[156] Sokolove J (2016). Impact of baseline anti-cyclic citrullinated peptide-2 antibody concentration on efficacy outcomes following treatment with subcutaneous abatacept or adalimumab: 2-year results from the AMPLE trial. Ann. Rheum. Dis..

[157] Cutolo M (2009). CTLA4-Ig interacts with cultured synovial macrophages from rheumatoid arthritis patients and downregulates cytokine production. Arthritis Res. Ther..

[158] Oi K (2019). Tumour necrosis factor α augments the inhibitory effects of CTLA‐4‐Ig on osteoclast generation from human monocytes via induction of CD80 expression. Clin. Exp. Immunol..

[159] Agemura T, Hasegawa T, Yari S, Kikuta J, Ishii M (2021). Arthritis-associated osteoclastogenic macrophages (AtoMs) participate in pathological bone erosion in rheumatoid arthritis. Immunol. Med..

[160] Wang W (2020). FOXM1/LINC00152 feedback loop regulates proliferation and apoptosis in rheumatoid arthritis fibroblast-like synoviocytes via Wnt/β-catenin signaling pathway. Biosci. Rep..

[161] Zeng R (2021). FOXM1 activates JAK1/STAT3 pathway in human osteoarthritis cartilage cell inflammatory reaction. Exp. Biol. Med..

[162] Weivoda MM, Lee SK, Monroe DG (2021). miRNAs in osteoclast biology. Bone.

[163] Alivernini S (2020). Distinct synovial tissue macrophage subsets regulate inflammation and remission in rheumatoid arthritis. Nat. Med..

[164] Chen Y (2022). Single-cell RNA landscape of the osteoimmunology microenvironment in periodontitis. Theranostics.

[165] Culemann S (2019). Locally renewing resident synovial macrophages provide a protective barrier for the joint. Nature.

